# *In vivo* time-harmonic ultrasound elastography of the human brain detects acute cerebral stiffness changes induced by intracranial pressure variations

**DOI:** 10.1038/s41598-018-36191-9

**Published:** 2018-12-17

**Authors:** Heiko Tzschätzsch, Bernhard Kreft, Felix Schrank, Judith Bergs, Jürgen Braun, Ingolf Sack

**Affiliations:** 1 Department of Radiology, , Charité - Universitätsmedizin Berlin, Berlin, Germany; 2 Institute of Medical Informatics, Charité - Universitätsmedizin Berlin, Berlin, Germany

## Abstract

Cerebral stiffness (CS) reflects the biophysical environment in which neurons grow and function. While long-term CS changes can occur in the course of chronic neurological disorders and aging, little is known about acute variations of CS induced by intracranial pressure variations. Current gold standard methods for CS and intracranial pressure such as magnetic resonance elastography and direct pressure recordings are either expensive and slow or invasive. The study objective was to develop a real-time method for *in vivo* CS measurement and to demonstrate its sensitivity to physiological aging and intracranial pressure variations induced by the Valsalva maneuver in healthy volunteers. We used trans-temporal ultrasound time-harmonic elastography (THE) with external shear-wave stimulation by continuous and superimposed vibrations in the frequency range from 27 to 56 Hz. Multifrequency wave inversion generated maps of shear wave speed (SWS) as a surrogate maker of CS. On average, cerebral SWS was 1.56 ± 0.08 m/s with a tendency to reduce with age (R = −0.76, p < 0.0001) while Valsalva maneuver induced an immediate stiffening of the brain as reflected by a 10.8 ± 2.5% increase (p < 0.0001) in SWS. Our results suggest that CS is tightly linked to intracranial pressure and might be used in the future as non-invasive surrogate marker for intracranial pressure, which otherwise requires invasive measurements.

## Introduction

Intracranial pressure (ICP) is one of the most important parameters for the assessment of the brain’s status with respect to blood perfusion, cerebrospinal fluid (CSF) drainage and autoregulation^1^. The ICP cannot be measured and monitored directly without skull opening, which limits its use for diagnostic purposes. Today, the gold standard for ICP measurement is intraventricular catheter while non-invasive tools such as transcranial Doppler ultrasound have been developed to assess ICP indirectly, e.g. by measurement of cerebrovascular reactivity (CVR)^2,3^.

CVR refers to the ability of the brain to regulate acute changes in ICP or blood perfusion by adaptation of vessel diameters in order to preserve healthy neurovascular coupling. Long-term CVR (in the order of minutes) can be probed by hypercapnia challenges^4^ and perfusion-sensitive magnetic resonance imaging (MRI) for the detection of cerebrovascular pathologies related to various diseases such as Multiple Sclerosis^5^. Dynamic CVR (in the order of seconds) can be induced by the Valsalva maneuver (VM) and quantified by transcranial Doppler ultrasound which is sensitive to pulsatile intracranial flow^6^. VM raises intrathoracic pressure which affects cerebral blood circulation and ICP^7^. Therefore, during VM the brain can be regarded as being in an intracranial hypertension state^8^.

Despite the tight coupling between pulsatile intracranial flow and ICP, transcranial Doppler ultrasound cannot measure ICP. Modeling ICP based on transcranial Doppler data relies on assumptions on tissue viscoelasticity^8^, which itself might change with alterations of ICP and CVR. Since pressure refers to a mechanical stress, a pressure-sensitive diagnostic method could be based on mechanical stress waves which can probe fluid-solid tissue interactions and pore pressure in multiphasic materials like brain tissue non-invasively^9–11^.

Currently, mechanical stress waves are exploited by magnetic resonance elastography (MRE) for *in vivo* measurement of brain viscoelasticity in a non-invasive manner^12,13^. MRE can produce maps of viscoelastic parameters of the brain similar to radiological images by externally inducing time-harmonic stress waves at audible frequencies between 20 and 60 Hz. *In vivo* viscoelastic parameters of the brain have been correlated with memory performance^14,15^, sex and age^16–19^, as well as with diseases such as frontotemporal dementia^20,21^, normal pressure hydrocephalus^22,23^, Alzheimer’s disease^24,25^, Multiple Sclerosis^26,27^ and Parkinson’s disease^28,29^.

However, MRE is currently limited by the relatively high variation of measured values among subjects^30^ and by examination times that cannot account for acute changes of brain physiology related to ICP, arterial pulsation or autoregulation^9^. Despite these limitations, several MRE studies have addressed effects of ICP on brain viscoelasticity by manipulating the cerebral venous return in healthy volunteers^31^, correlating MRE with perfusion MRI within deep gray matter regions^32^ and hypercapnia^33^ or measuring ICP and MRE in a swine model with implanted cerebral catheters^34^.

In contrast, ultrasound elastography, which is routinely used for diagnostic examinations of hepatic fibrosis or tumors, has high temporal resolution but is limited for investigations of the human brain due to the thickness of the skull^35–37^. Ultrasound based cerebral elastography has recently been applied to neonates via the fontanelles without the obstruction caused by the skull^38^. Thalamus and periventricular white matter were found to be significantly lower in stiffness in the preterm group compared with the term group^39^. In adults, ultrasound elastography was successfully applied after skull opening for the assessment of neurological tumors and for reporting healthy-tissue values in agreement to MRE^40^. However, a recent study using acoustic radiation force impulse (ARFI) ultrasound elastography through the temples of healthy adults could not reproduce these findings and reported lower values than those known from the literature^41^. A major limitation of non-invasive transcranial ultrasound elastography is insufficient ARFI-intensity penetrating the skull for transient shear wave stimulation as commonly used in ultrasound elastography^10^.

In previous work, we have developed time-harmonic elastography (THE) based on ultrasound and continuous vibrations produced by a loudspeaker integrated in the patient bed^10^. The frequency range of vibrations in THE is between 27 and 56 Hz, similar to MRE, making it possible to combine fast ultrasound motion detection with the mechanical excitation of deep tissues such as native kidneys^42^, the liver in obese patients^43^, the spleen^44^ or the heart^45^. THE may also confer a unique tool for rapid transcranial viscoelasticity measurements by ultrasound and assessment of CVR-related stiffness changes in the brain.

In this work, our hypothesis is that cerebral THE can measure brain stiffness linked to ICP changes and CVR due to the underlying solid-fluid interactions in brain tissue^11,46–48^. To test this hypothesis, we developed THE for cerebral stiffness (CS) measurements, tested its reproducibility, analyzed baseline values in healthy volunteers covering a larger age range, and applied the new method to volunteers who repeatedly underwent VM.

## Material and Methods

### Study cohort

The study protocol conformed to the guidelines of the Declaration of Helsinki and was approved by the institutional review board of Charité – Universitätsmedizin Berlin (EA1/063/18). Informed written consent was obtained from all study participants. None of the study participants had any history of neurological disease including psychiatric disorders and/or history of major head injury. A total of 26 healthy volunteers (9 females, mean age: 39.9 ± 18.6 years, age range: 21 to 86 years) were investigated. Information about the heart rate and blood pressure of every study participant can be found in the Supplementary Table 1.

10 subjects (4 females, mean age: 29.7 ± 8.7 years, age range 25 to 57 years) were scanned four times: twice for test-retest reproducibility measurements by two different operators. Both operators observed the same brain region through the right temporal bone window as described below. Between the test-retest measurements, the volunteers were asked to stand up and move around.

### Cerebral THE

**Figure 1:**
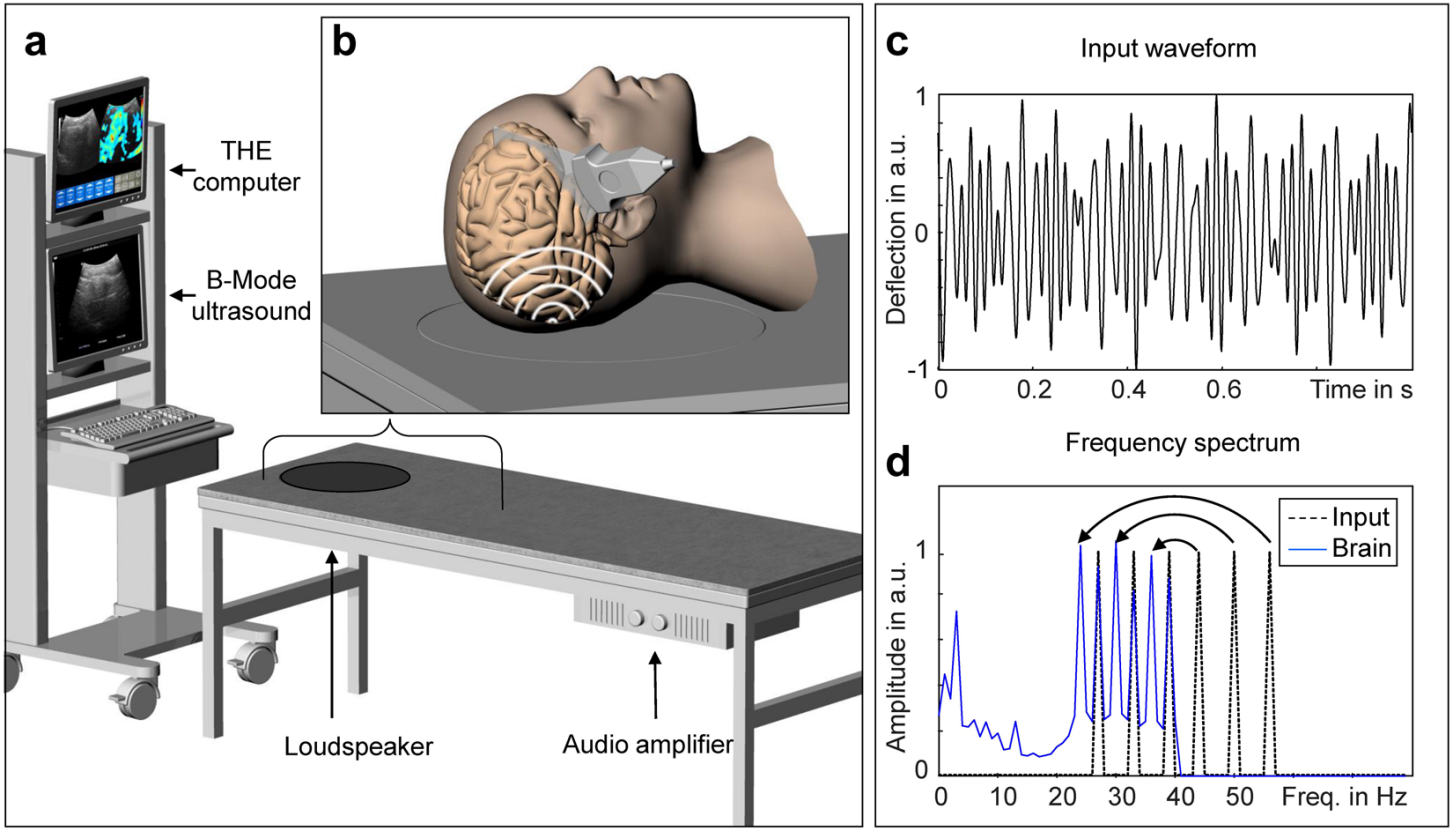
Technical setup of cerebral time-harmonic elastography (THE) by transcranial ultrasound. (**a**) Conventional B-mode ultrasound scanner connected to an elastography computer for real-time data processing and a vibration bed which includes a loudspeaker and audio amplifier for external wave stimulation. (**b**) Head position on the bed, which is centered on a circular rubber mat mounted onto the loudspeaker membrane beneath. The transducer was placed on the temporal region of the volunteer’s skull to select a transtemporal cranial window suitable to guide elastography measurements through the temporal lobe up to a depth of approx. 8 cm. (**c**) Waveform fed into the loudspeaker consisting of six frequencies of 27, 33, 39, 44, 50, 56 Hz. (**d**) Power spectrum of the multifrequency vibration signal from the loudspeaker input (dashed line) and measured in a volunteer’s brain (blue line). The curved arrows indicate aliasing of higher frequencies (>40 Hz) due to the low frame rate of 80 Hz.

**Figure 2:**
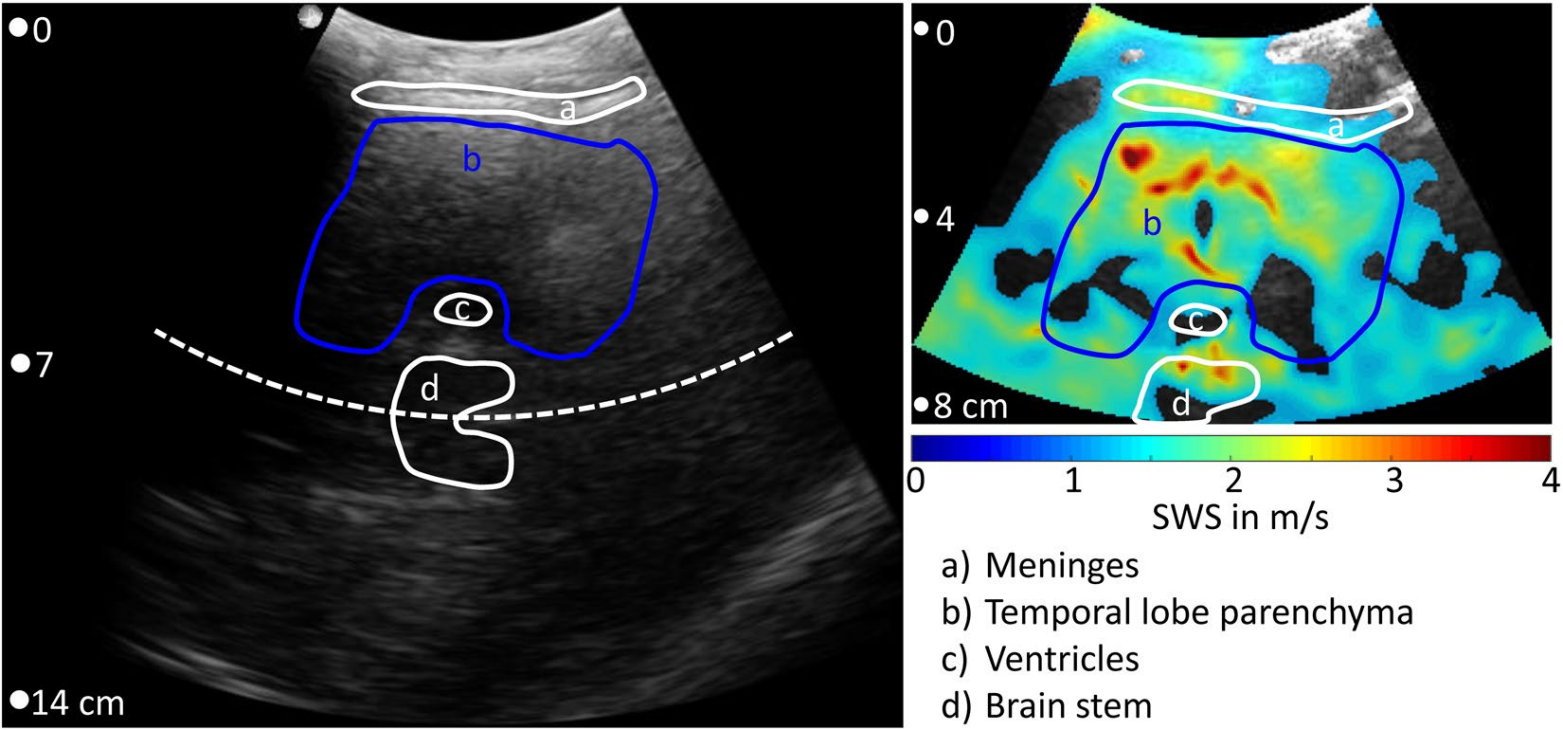
Transtemporal B-mode (left panel) and corresponding THE-SWS map (colored overlay in the right panel). The field-of-view in elastography measurements covered only 8 cm depth in order to accommodate a frame rate of 80 Hz. Anatomical regions are demarcated by lines which encompass the landmarks visible in the B-mode. The regions-of-interest (ROI) for interrogating brain stiffness was placed within the temporal lobe (blue lines). As explained in the Methods section, wave speed values below 1.2 m/s were neglected yielding holes in the colored SWS overlay. Note, B-mode and elastogram result from different scans and show slightly varying anatomical regions. Additional cases can be found in the supplementary material.

Time-harmonic ultrasound elastography (THE) with 2D multifrequency shear wave speed (SWS) compounding for real-time elastogram display was introduced in Tzschätzsch *et al*.^44^. The setup consists of (1) a vibration bed for shear wave excitation, (2) a standard B-mode ultrasound scanner for shear wave acquisition, and (3) an elastography computer for shear wave evaluation and visualization (Fig. 1a). The vibration bed was customized by a loudspeaker (Raptor 12, Monacor International, Bremen, Germany) and audio amplifier (PLX 3402, QSC Audio Products, Costa Mesa, CA USA) both mounted onto a standard patient bed (ULI 6000 PLX 3402, AGA Sanitätsartikel GmbH, Löhne, Germany). Different from our previous THE setup used in the abdomen^44^, the loudspeaker for brain stimulations was placed underneath the head position (Fig. 1b). The waveform fed into the amplifier consisted of six frequencies of 27, 33, 39, 44, 50, 56 Hz with equal amplitudes as shown in Fig. 1c,d. The amplitudes were equal for all frequencies since no frequency-dependent wave attenuation could be found in the brain (see Fig. 1d); unlike amplitudes found in liver THE studies^44^. Of note, the range of frequencies is similar to what has been established in time-harmonic elastography by MRE^9^. This range represents a viable compromise between the two margins of low wavenumbers (at low frequencies) and high damping of the waves (at higher frequencies). As explained below, the specific frequencies in THE were chosen since they do not overlap in the spectrum when aliased due to the low sampling rate of the clinical ultrasound scanner of 80 Hz. The ultrasound device (SonixMDP, Ultrasonix, Scottsdale AZ, USA) was equipped with a convex transducer (C5–2/60) of 2 MHz frequency. The transducer position at the right temples of the volunteers was real-time adjusted to identify all the relevant landmarks of the temporal lobe including the inner cerebrospinal fluid spaces, meninges, and brain stem through an angulated view with up to 8 cm penetration depth (Fig. 1b). Figure 2 shows a representative B-mode image with demarcated landmarks used for guidance of THE in a volunteer. Additional cases can be found in the Supplementary Fig. 1.

**Figure 3:**
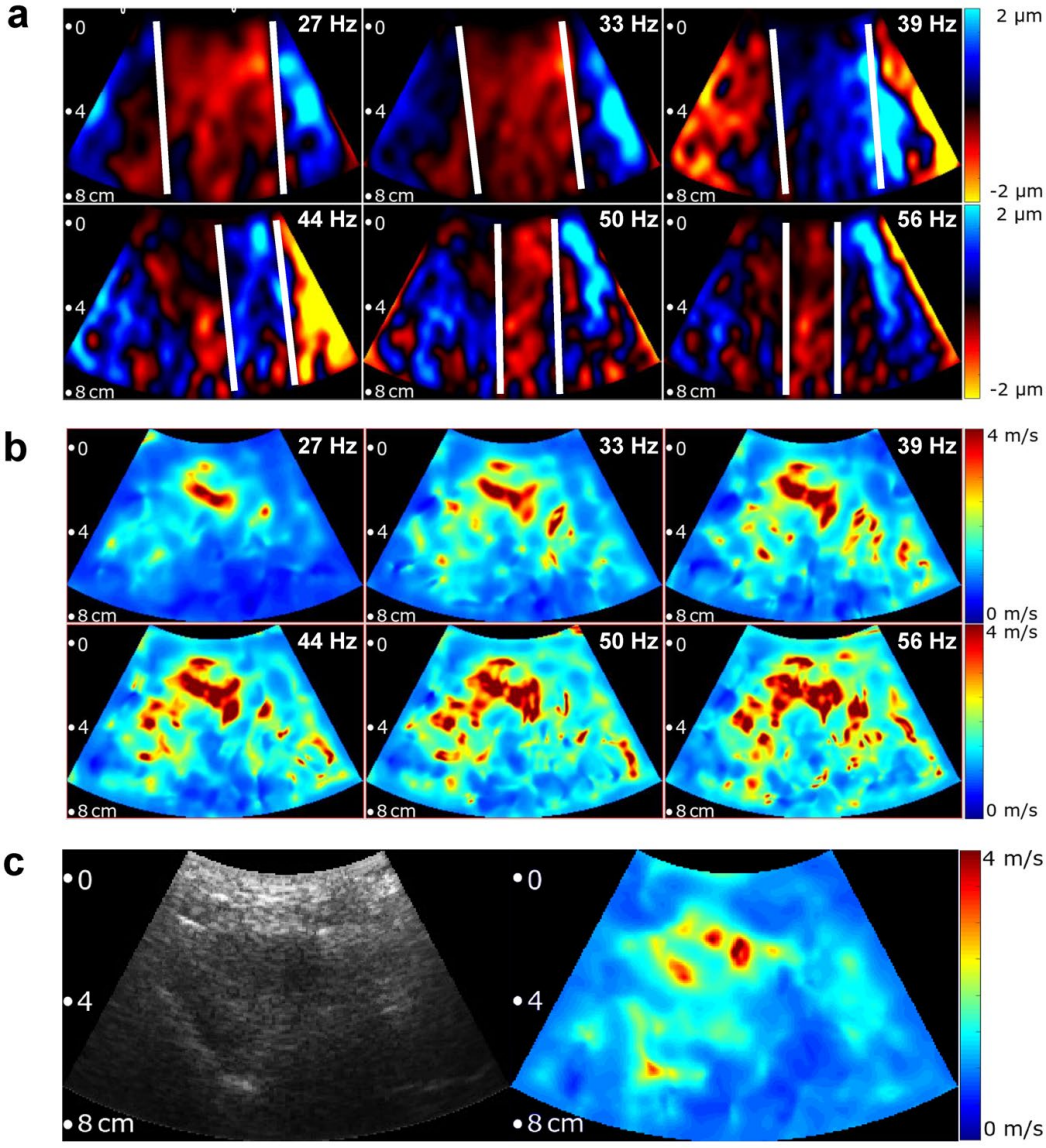
Multifrequency SWS compounding. (**a**) Raw wave images at six excitation frequencies. White lines demarcate wave fronts to illustrate the approximate lengths of half-waves. (**b**) Single-frequency elastograms (SWS maps) obtained after wave-gradient based inversion^44^. (**c**) Representative B-mode image (left hand side) and final elastograms (SWS map, right hand side) which was generated from the six single-frequency SWS maps shown in (**b**).

Ultrasound raw data were acquired over one second with an 80 Hz frame rate. As a result of the low frame rate, frequencies higher than 40 Hz (i.e. 44, 50 and 56 Hz) are mapped onto aliased frequencies (i.e. 36, 30 and 24 Hz) according to the principle of controlled aliasing^44^, as indicated in Fig. 1d and further explained in the supplementary material. Acquired data were continuously transferred to the elastography PC in real-time for online processing and visualization. Shear wave post-processing included the following steps: (1) motion estimation for deriving the axial tissue displacement, (2) decomposition of the 6 vibration frequencies via Fourier transformation, (3) multifrequency wave-number reconstruction followed by inversion to generate compound shear wave speed (SWS) maps^50^ (see supplementary material) and, (4) online display of B-mode and SWS maps for regional averaging as illustrated in Fig. 3. Regions-of-interest (ROI) were manually selected to delineate brain tissue within the temporal lobe excluding CSF and dura mater as illustrated in Fig. 2 and Supplementary Fig. 1. Within the selected ROI, all SWS values below 1.2 m/s were automatically discarded while all other pixel values were considered reliable and averaged for further analysis. For measurement of reference values and to determine an age effect, each experiment was repeated twenty times and averaged in order to account for minimal variations of transducer position and head position with respect to the loudspeaker membrane. Total processing time for data acquired within 1 s was approximately 3 s. In addition to this ‘normal-mode THE’, we used ‘temporally-resolved THE’ for testing the dynamic change of SWS in response to VM. THE with temporal resolution required 7 s continuous data acquisition and off-line post processing. Therefore, a temporal Hilbert transform was applied to the motion data prior to frequency-filtering using a Gaussian bandpass with σ = 0.95 Hz yielding complex-valued wave images with 1/80 Hz time resolution. The further processing of the complex wave images was identical to ‘normal mode’ processing as described in the supplementary material and in our previous work^50^.

In the literature, tissue stiffness is reported by shear modulus, Young’s modulus or SWS^10^. Without conversion, these quantities can be used for addressing relative property changes related to tissue stiffness. Henceforth, we refer to cerebral stiffness (CS) whenever relative values are discussed and compared to the literature while the physically well-defined quantity SWS (in m/s) will be used when reporting values.

### Study protocol for the Valsalva maneuver

The Valsalva maneuver is defined as maximum voluntary abdominal force induced by abdominal muscle contraction during breath hold at inspiration identical to the maneuver defined in Ipek-Ugay *et al*.^49^. First, temporally resolved THE was applied in three subjects (2 females, 25, 28 and 33 years old) to investigate the time-course of cerebral stiffness changes. As compared to ‘normal-mode THE’, ‘ temporally-resolved THE’ is computationally expensive and requires off-line data processing. Five different VM states of approx. 10 seconds each were defined during which the data were continuously sampled in the time-resolved THE mode. VM states were defined as (1) pre-VM baseline, (2) transition from baseline to VM, (3) full VM, (4) transition from VM to post-VM relaxation, and (5) post-VM relaxation^49^. In a second experiment, 10 subjects (4 females, mean age: 32.1 ± 7.1 years, age range 26 to 47 years) were scanned 10 times before and 10 times during VM in an interleaved fashion using THE in the normal mode (not time resolved).

### Statistical analysis

Reproducibility was tested by intraclass correlation coefficients (ICC), both accounting for intra- and interobserver variability defined according to^51^ as

1 $$IC{C}_{{\rm{intra}}}=S{D}_{{\rm{group}}}^{2}/(S{D}_{{\rm{group}}}^{2}+S{D}_{{\rm{subject}}}^{2})$$

2 $$IC{C}_{{\rm{inter}}}=S{D}_{{\rm{group}}}^{2}/(S{D}_{{\rm{group}}}^{2}+S{D}_{{\rm{subject}}}^{2}+S{D}_{{\rm{operator}}}^{2})$$

with $S{D}_{{\rm{group}}}$, $S{D}_{{\rm{subject}}}$, and $S{D}_{{\rm{operator}}}$ denoting standard derivations among subjects, within measurements in each subject by the same operator and between measurements by different operators. The significance of SWS changes due to VM was tested by a paired t-test. Correlation analysis of SWS with age was performed by Pearson’s linear correlation coefficient, p-values < 0.05 were considered significant. Box and whisker plots were used for visualization of group data by means of lower quartile, median, and upper quartile values, as well as the extent of all data including outliers.

## Results

Figure 3 shows representative wave images and SWS maps of a volunteer’s brain acquired at 6 different frequencies. Frequency-resolved SWS-maps reveal more artifacts than compound maps generated by multifrequency inversion, demonstrating the advantage of multifrequency SWS compounding above single-frequency THE of the brain which mirrors similar results obtained in abdominal organs^44,50^. Figure 3b also demonstrates an increasing intensity of SWS features with increasing frequency due to the well-known viscoelastic dispersion of brain tissue^52^ which is lost by multifrequency compounding. Size and positions of features in SWS maps correlated only marginally with anatomical landmarks in the B-mode images (Fig. 2 and Supplementary Fig. 1). Such details of stiffness (with an approx. resolution of 2 to 3 mm) were not further analyzed in this study due to the overall limited image quality of transcranial ultrasound (see B-mode in Fig. 2 and Supplementary Fig. 1).

**Figure 4:**
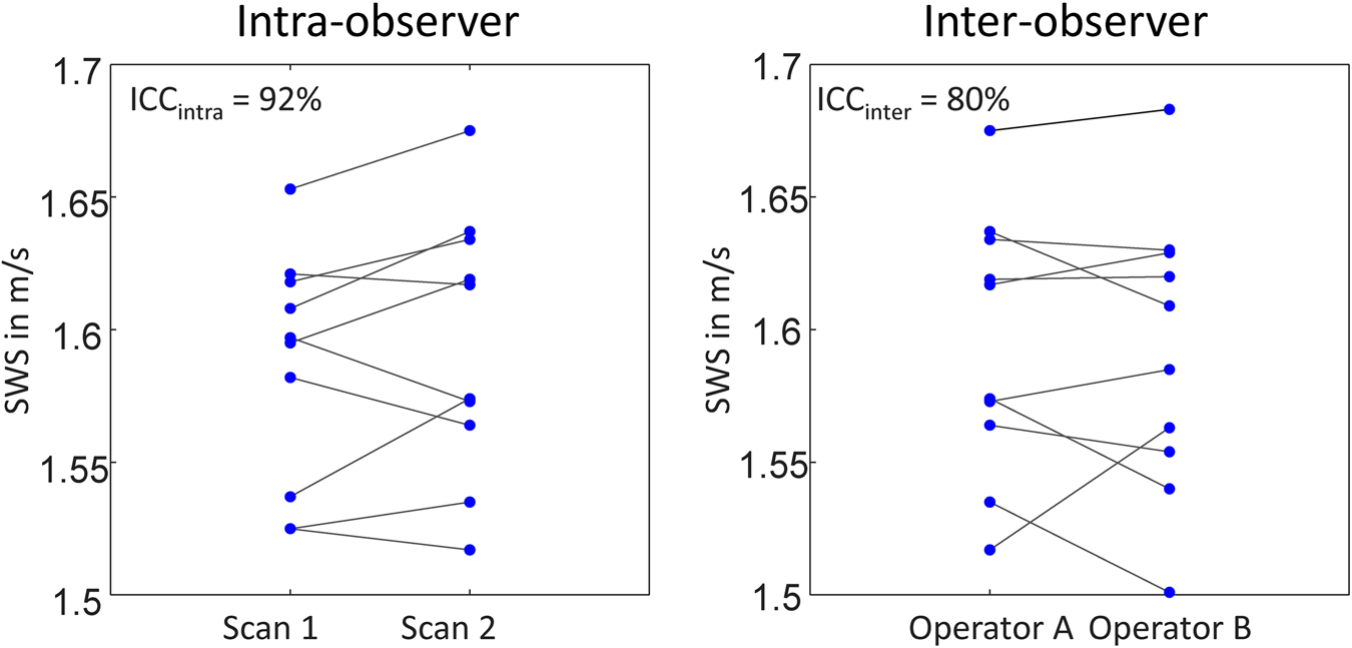
Reproducibility of SWS measured by transcranial SWS. Two operators have performed two scans in ten subjects prior (scan 1) and after position changes (scan 2) demonstrating the good reproducibility of the method.

Instead, reproducible SWS values were obtained by collapsing all spatial information into a single value by averaging SWS within the ROI as described above. The reproducibility is demonstrated by test-retest analysis shown in Fig. 4. Mean SWS values, including scan 1 and 2, were identical between operator A and B (1.59 ± 0.06 m/s). The same applies to the mean difference in SWS between scan 1 and scan 2 (Δ*SWS* = |*SWS*_scan1_ − *SWS*_scan2_|), which is 0.02 ± 0.01 m/s for both operators. $IC{C}_{{\rm{intra}}}$ and standard deviation were 91.6% and 0.03 m/s for operator A and 90.8% and 0.03 m/s for operator B. $IC{C}_{{\rm{inter}}}$ was 80.0% with a standard deviation of 0.02 m/s. All values measured by operator 1 and 2 in both examinations are shown in Fig. 4.

**Figure 5:**
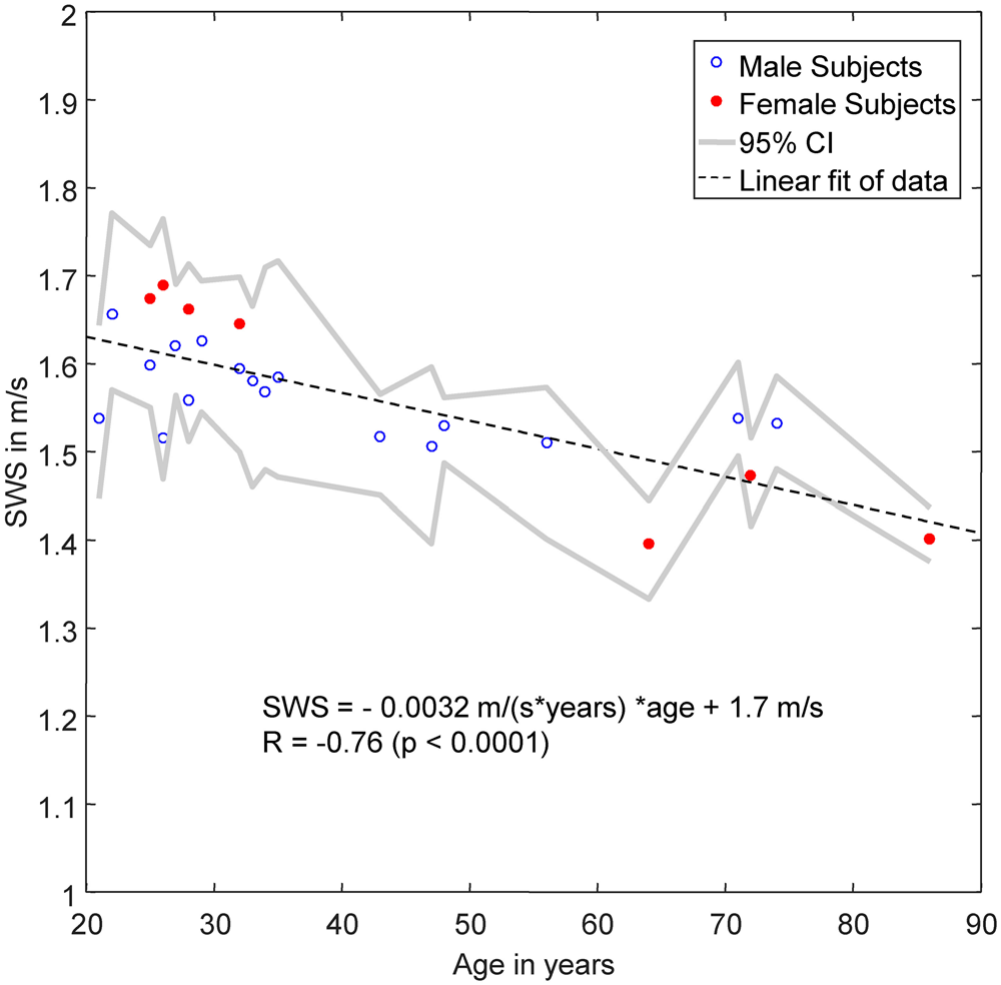
Baseline SWS in healthy subjects over years of age. Gray lines demarcate 95% confidence intervals (CI). The linear regression line (dashed line) was fitted to pooled data without consideration of sex differences.

Figure 5 shows reference SWS values in an age range between 21 and 86 years. Mean SWS was measured with 1.56 ± 0.08 m/s (corresponding to the age of 41 years) with tendency towards lower values with increasing age (−0.003 m/s or −0.2% per year R = −0.76, p < 0.0001).

**Figure 6:**
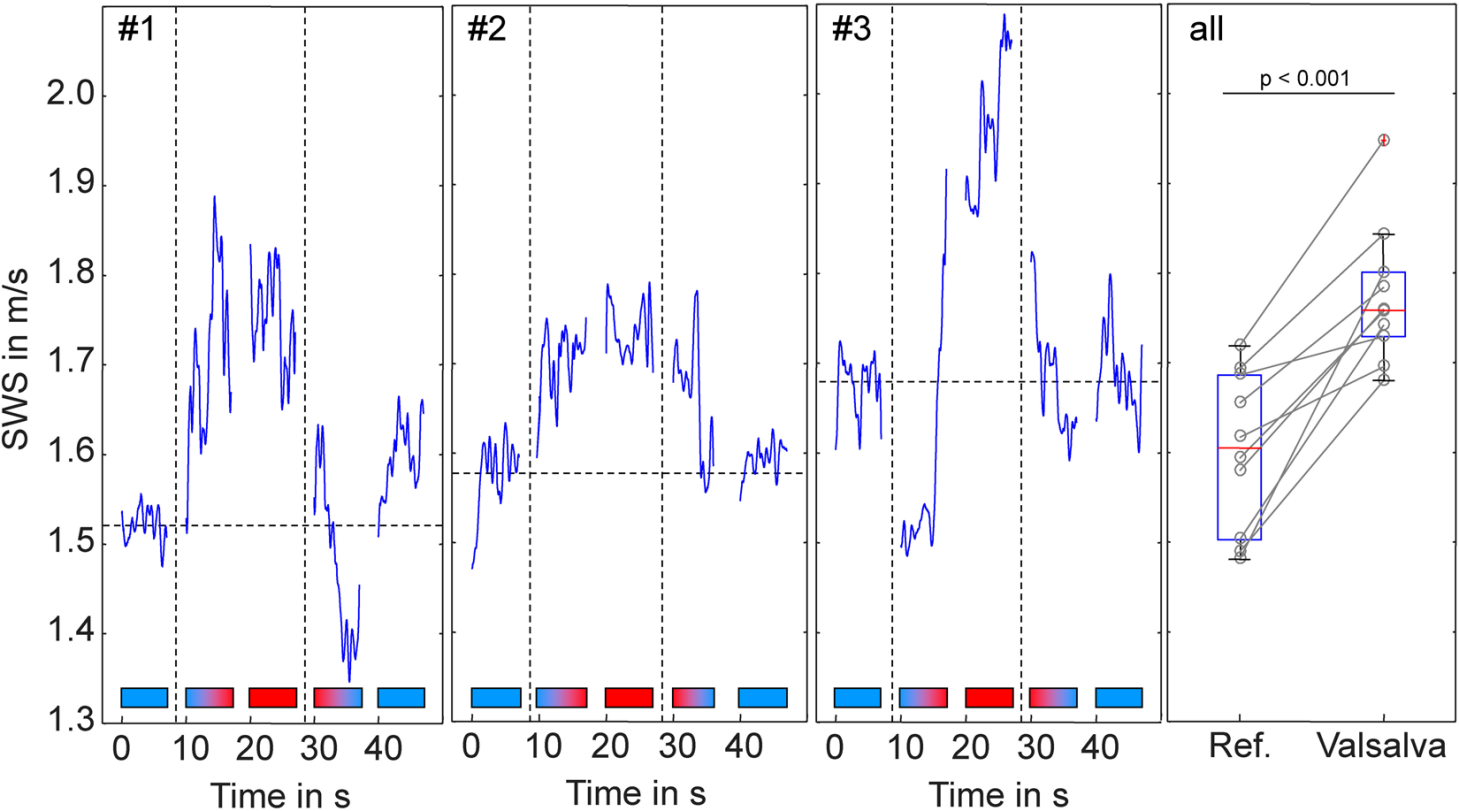
Effect of Valsalva maneuver (VM) on brain stiffness (shear wave speed, SWS in m/s). Three volunteers (#1, #2, #3) were investigated by temporally resolved THE with a resolution of 12.5 ms showing the time course of stiffness changes under different VM-states. Horizontal color bars illustrate VM states (blue: resting state, red: VM, blue-red: transition from rest to VM, red-blue: transition from VM to rest). The panel on the right-hand-side (all) shows the increase in stiffness in 10 volunteers performing repeated VM maneuvers with short interleaved resting periods.

Figure 6 demonstrates the influence of VM-induced ICP changes on cerebral SWS. Our temporally-resolved THE protocol (offline data processing) in three volunteers (#1 to #3) revealed the instantaneous response of SWS to VM. The five measurement intervals during state 1 to state 5 are demarcated by horizontal color bars in which blue color indicates relaxation and red color indicates maximum abdominal muscle contraction during breath-hold yielding a strong VM paradigm. During the transition phases (state 2 and 4) SWS visibly increased or decreased corresponding to the direction of the applied maneuver while the variation of values was lower during phases of constant conditions (state 1, 3 and 5). Overall, these results suggest that SWS can significantly change within short breath hold intervals of approx. 10 seconds. Therefore, THE was repeatedly applied during relaxation intervals followed by 10-s VM intervals yielding the averaged group values shown in Fig. 6 on the right hand side. On average, VM induced an SWS increase of 10.8 ± 2.5% (p < 0.0001).

## Discussion

To our knowledge, this is the first report on brain shear stiffness measurements by *in vivo* transcranial ultrasound based on time-harmonic elastography in humans. The proposed THE modality addresses a methodological gap which exists between real-time intracranial ultrasound and MR elastography. While the former uniquely allows cerebrovascular reactivity (CVR) assessment by fast Doppler flow measurements, without any direct tissue stiffness quantification, the latter (MRE) can measure *in vivo* brain stiffness but is limited due to long scanning times impeding the observation of short-term CS changes.

Our values are in agreement with those reported in *in vivo* open-skull shear wave ultrasound elastography studies, which found for normal brain tissue 1.6 ± 0.5 m/s CS^40^. Our group mean SWS of 1.56 ± 0.08 m/s can be converted to 2.44 ± 0.25 kPa shear modulus employing the elastic model (CS [in kPa] = SWS²·ρ, assuming a density ρ of 1000 kg/m^3^ for brain tissue), which well agrees with MRE at 50 and 60 Hz (see table 3 in Hiscox *et al*.^12^). Also, the observed decline in CS with age reproduces previous findings by MRE. We and others have observed a linear decline in whole-brain shear modulus in the order of 0.5% per year^16–18^. Our present study converted to shear modulus suggests a similar annual rate of change of −0.4%. This agreement in effect sizes observed by different modalities is encouraging and indicates the robustness of cerebral THE. However, future studies should address the direct comparison of CS values between THE and MRE in individuals in order to gain more insight into the variability of *in vivo* CS in the range of physiological variations.

One intriguing finding of our study is the increase of CS by the Valsalva maneuver, suggesting that neural shear stiffness contributes to cerebrovascular reactivity. VM is a suitable maneuver to study CVR since it induces a short-term increase in ICP^53^ in the order of 5.7 mmHg^54^, which is approx. 40% ICP change compared to resting conditions. Our mean VM-induced CS increase of approx. 10% indicates that micro flow in capillaries and CSF cavities effectively communicates fluid pressure changes into the global shear modulus as predicted for multiphasic poroelastic materials^11,46–48^. Such mechanism might in the future allow using CS as a surrogate marker for relative ICP changes while absolute CS values clearly depend on the contribution of multiple mechanically active networks in the brain including neurons, oligodendrocytes, astrocytes and the vascular tree. Nonetheless, rapid measurements of CS during physiological maneuvers, intrinsic arterial pulsation or perfusion changes, might help to assess the individual capacity of regulating ICP alterations for neurological diagnoses.

A current limitation of cerebral THE is its low resolution of details, which is shared with the limited resolution and contrast of normal transcranial B-mode ultrasound. However, despite this limitation, transcranial ultrasound is routinely used in the clinic for Doppler flow measurements and CVR assessment. Moreover, in our proof-of-concept study the used B-mode ultrasound system was not further optimized for imaging examinations of the brain. Also, the spectrum of frequencies used for brain stimulations was adopted from THE in abdominal organs and based on experiences made by cerebral MRE. Nevertheless, a setup optimized for brain tissue could potentially yield SWS maps with more anatomical details such as gray and white matter interfaces. For example, contrast agents could be applied in order to improve the echogenicity of highly vascularized brain matter such as cortical or deep gray matter^37^ and to better resolve the anatomy of brain viscoelastic properties by ultrasound and time harmonic waves.

In summary, transcranial THE is feasible for rapid noninvasive brain stiffness measurements.

The acquired values in healthy adults with a mean of 1.56 ± 0.08 m/s including a slight reduction with age in the order of 0.2% per year are in agreement with literature studies based on interventional ultrasound elastography and cerebral MRE. It was demonstrated that THE-measured brain stiffness increases due to the Valsalva maneuver by approx. 10% rendering THE a promising modality for noninvasively identifying acute changes of ICP and intracranial perfusion. In the future, THE could be implemented as a diagnostic tool in the clinical workflow for monitoring pathological ICP alterations.

## Electronic supplementary material

Supplementary material
