# Supplementary material for: In vivo time-harmonic ultrasound elastography of the human brain detects acute cerebral stiffness changes induced by intracranial pressure variations
Source: Sci Rep. 2018 Dec 17;8:17888. doi: 10.1038/s41598-018-36191-9 (PMC6297160; doi:10.1038/s41598-018-36191-9)
Supplement: Supplementary file 1 — Supplementary material [file 41598_2018_36191_MOESM1_ESM.docx]

**Supplementary material: In vivo time-harmonic ultrasound elastography of the human brain detects acute cerebral stiffness changes induced by intracranial pressure variations.**

Heiko Tzschätzsch^1^, Bernhard Kreft^1^, Felix Schrank^1^, Judith Bergs^1^, Jürgen Braun^2^, Ingolf Sack^1^

^1^Department of Radiology, Charité - Universitätsmedizin Berlin, Berlin, Germany

^2^Institute of Medical Informatics, Charité - Universitätsmedizin Berlin, Berlin, Germany

1. **Description of the shear wave speed (SWS) reconstruction method**
2. **Supplementary figure 1A-C: Three additional cases of the display cerebral THE in B-mode and SWS maps.**
3. **Supplementary table 1: Description and measured SWS values of all volunteers.**

**Description of the shear wave speed (SWS) reconstruction method**

The method is described in detail in Tzschätzsch et. al^1^. In the following, a brief explanation of the most crucial steps of SWS reconstruction is provided. First, complex-valued in-phase quadrature (IQ) data are generated based on the Hilbert transformation of the raw radio-frequency (RF) data. Axial displacement $d_{i}$ is then estimated by calculating the phase shift between adjacent frames (*i*, *i*+1) in the IQ-data as proposed by:

$$d_{i}\propto\text{arg}\left( {IQ}_{i}\bar{{IQ}_{i+1}} \right)$$

The resulting time-resolved displacement field is decomposed by temporal Fourier transform into the complex wave fields for every externally induced vibration frequency (*f* = 27, 33, 39, 44, 50, and 56 Hz). Note, with a low frame-rate such as applied in our experiments of $f_{R}=$ 80 Hz, all higher vibration frequencies ($f>f_{R}/2$, here: 44, 50 and 56 Hz) are beyond the Nyquist limit and appear at aliased spectral positions ($f_{R}-f$, for $f>f_{R}/2$, here: 36, 30 and 24 Hz). Thus, we selected the complex-valued wave data from 24, 27, 30, 33, 36 and 39 Hz corresponding to the unaliased mechanical vibration frequencies of *f* = 56, 27, 50, 33, 44 and 39 Hz, respectively. Henceforth, this stroboscopic sampling of high vibration frequencies by relatively low frame rates is referred to as ‘controlled aliasing’.

The obtained complex wave images at vibration frequency usually show a lateral phase shift, which is artificially introduced whenever adjacent lines are acquired while the wave continues to propagate. The acquisition delay between adjacent lines is given by $\tau=1/\left( N_{LoS}{\cdot f}_{R} \right)$ (with $N_{LoS}$ denoting the number of lines-of-sight) and is used for correction of the lateral phase shift with $-2\pi\cdot f\cdot\tau$. A spatial band-pass-filter is further applied to every complex wave image at vibration frequency in order to suppress noise and unwanted motion. The kernel of this bandpass filter,

$$\text{ξ = }{\left| k \right|\cdot\exp\left( -\frac{1}{2}\left[ \frac{\left| k \right|}{\sigma} \right]^{2} \right),}$$

(with $k$, denoting wave numbers) was adapted to frequency by $\sigma=$ [255, 282, 307, 326, 347, 367] rad/m for $f=$[27, 33, 39, 44, 50 ,56] Hz. We note that this filter is slightly frequency dependent in the way that for lower vibration frequencies the filter maximum is centered at smaller wave numbers compared to higher frequencies.

After band-pass filtering, the remaining shear-wave field still reflects interferences of waves from multiple directions. To decompose this reverberant time-harmonic shear-wave field into unidirectional plane waves, such as $u=A \exp(-i k r)$, a directional filter^2^ with eight directions is employed as further specified in^3^. In the next step, the reconstruction of wave numbers $k$ for every shear wave direction and every vibration frequency is derived from complex shear waves $u$ employing the phase gradient method:

$$k=\left\| \nabla\text{arg}\left( u \right) \right\|$$

SWS is calculated by inversion of wave numbers $k$ for every wave direction and driving frequency. Finally, all SWS images are combined into a compound SWS map by amplitude-weighted averaging as explained in Tzschätzsch et al^1^. The weighted averaging for SWS compounding limits the effect of noise and low shear-wave amplitudes (e.g. from wave directions which do not reflect true shear-wave propagation) on our SWS maps.

**References**

1 Tzschätzsch, H. *et al.* Two-Dimensional Time-Harmonic Elastography of the Human Liver and Spleen. *Ultrasound Med Biol* **42**, 2562-2571 (2016).

2 Manduca, A., Lake, D. S., Kruse, S. A. & Ehman, R. L. Spatio-temporal directional filtering for improved inversion of MR elastography images. *Med Image Anal* **7**, 465-473 (2003).

3 Tzschätzsch, H. *et al.* Tomoelastography by multifrequency wave number recovery from time-harmonic propagating shear waves. *Med Image Anal* **30**, 1-10, doi:10.1016/j.media.2016.01.001 (2016).

**Supplementary figure 1A**


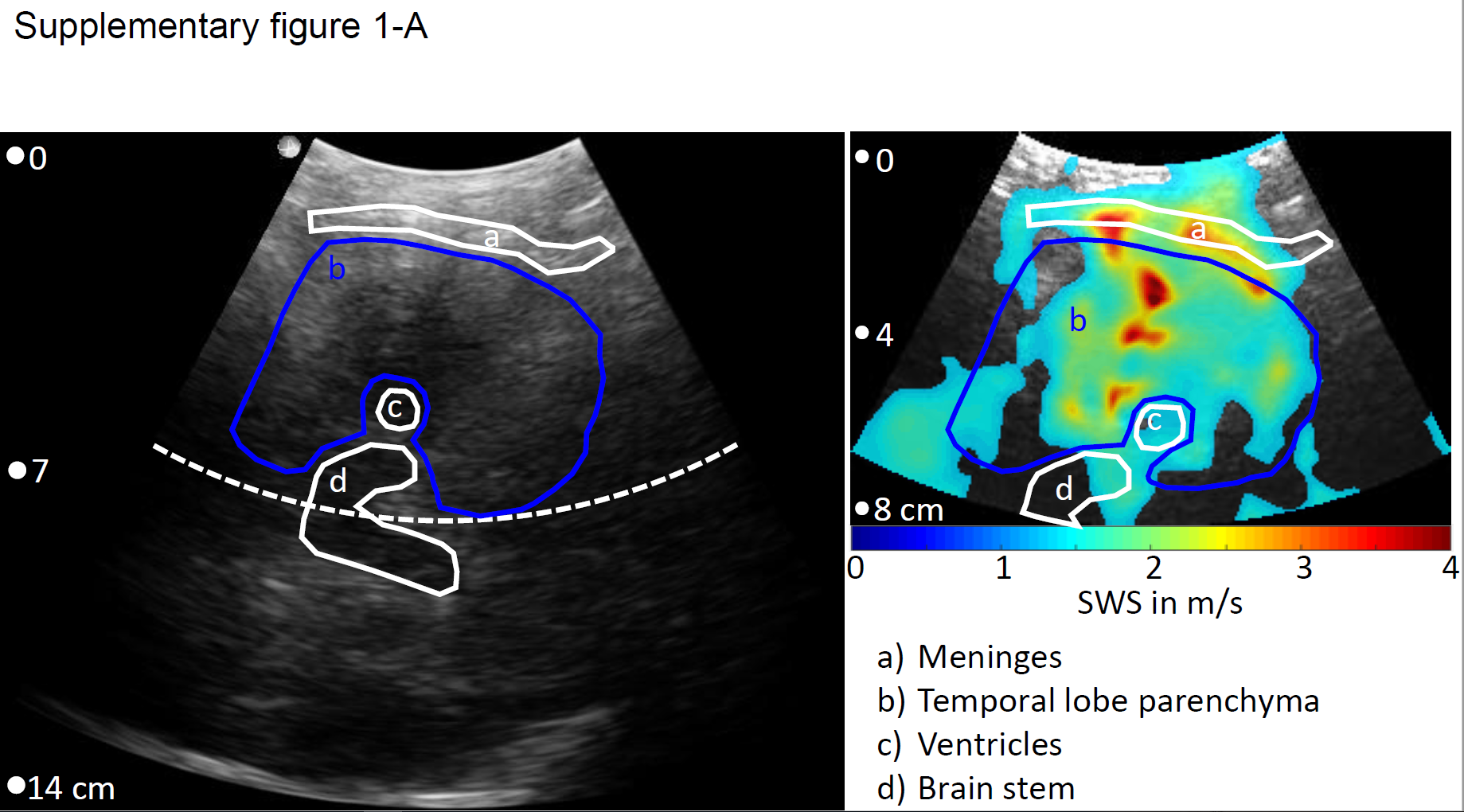


**Supplementary figure 1B**


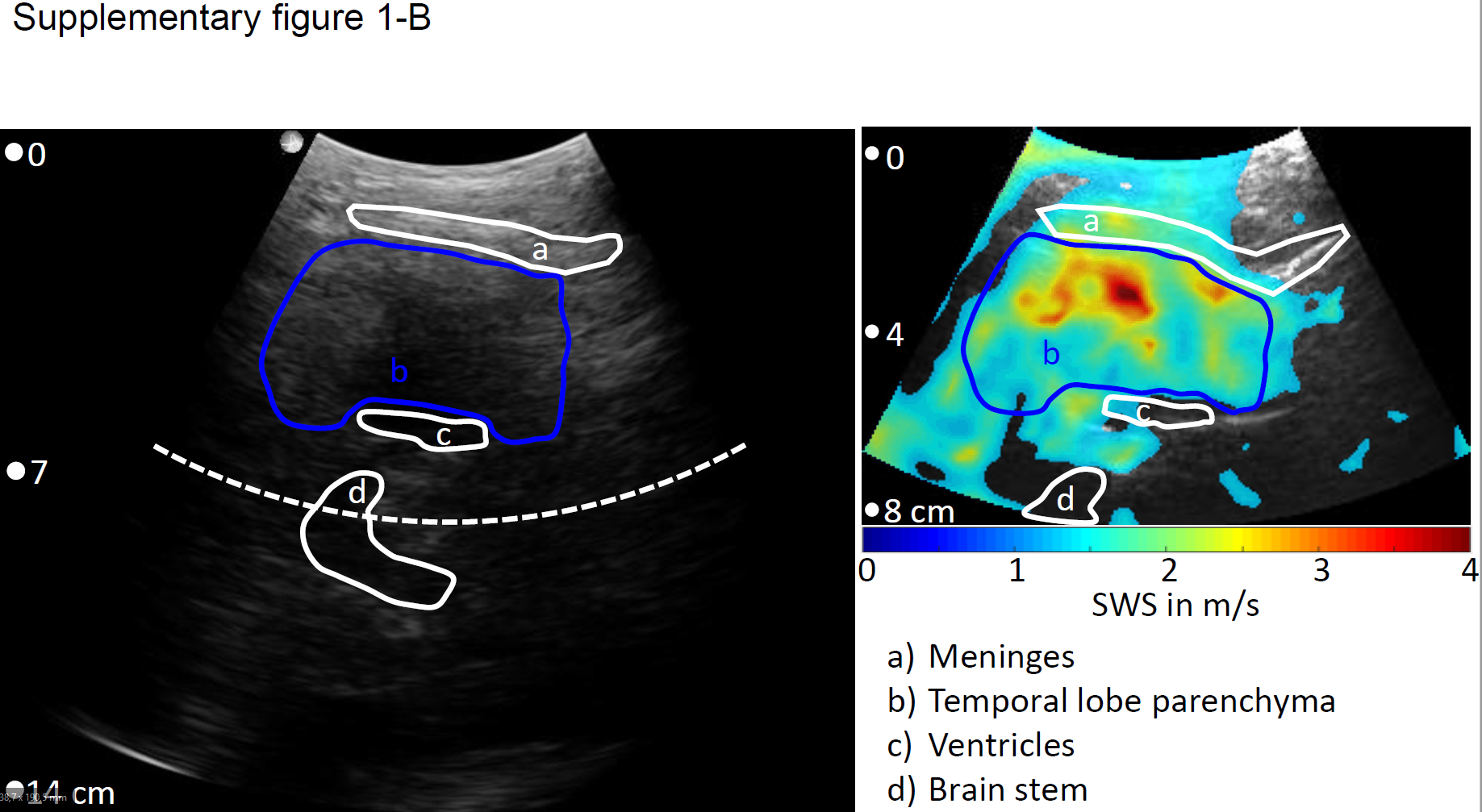


**Supplementary figure 1C**


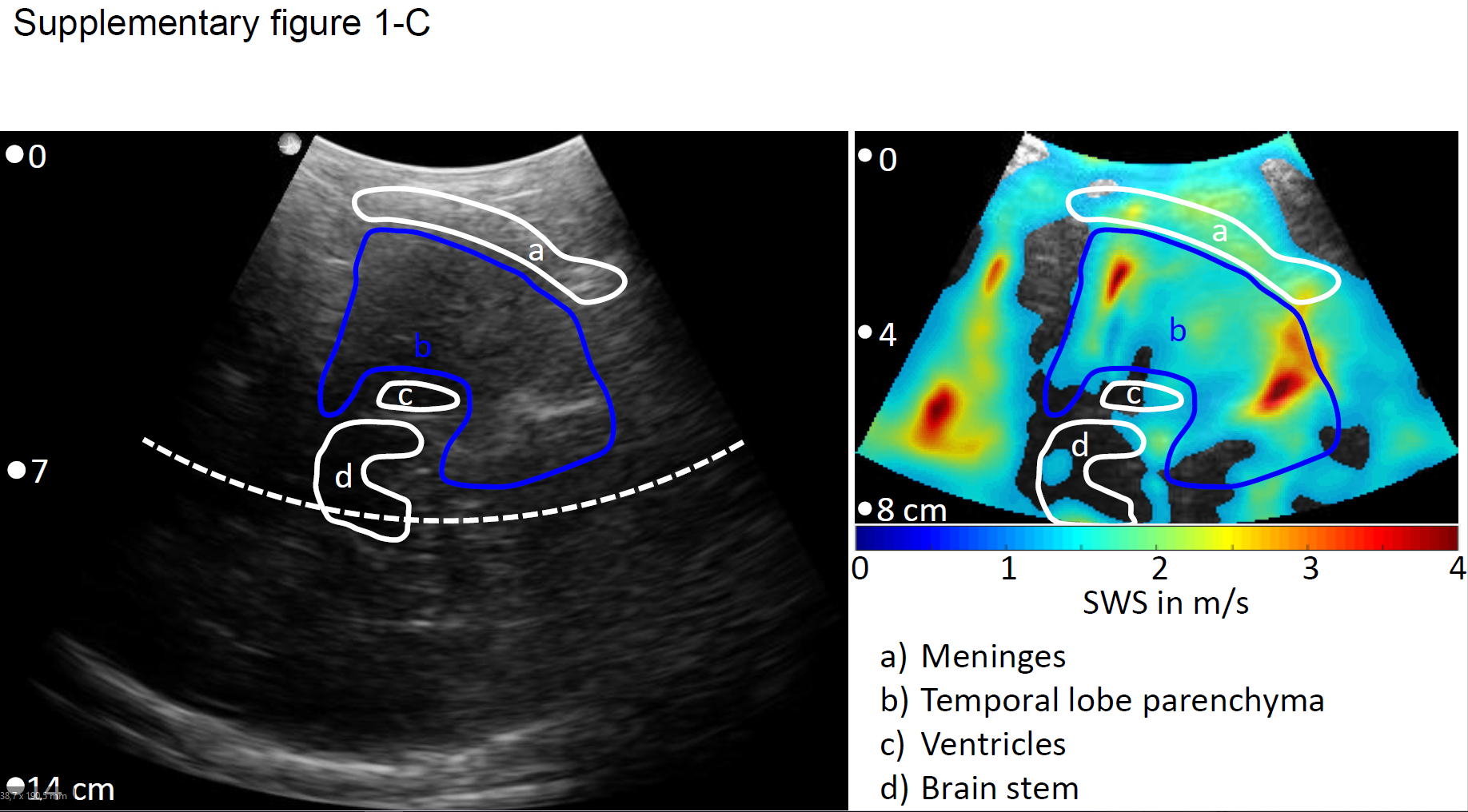
**Supplementary figures 1A-C:** Three cases of cerebral THE corresponding to figure 2 in the main document. Transtemporal B-mode images are shown in the panels on the left-hand side together with corresponding THE-SWS map (colored overlays) on the right-hand side. The field-of-view in elastography measurements covered only 8 cm depth in order to accommodate a sampling rate of 80 Hz. Anatomical regions are demarcated by lines which encompass the landmarks visible in the B-mode. The regions-of-interest (ROI) for interrogating brain stiffness was placed within the temporal lobe (blue lines). As explained in the Methods section, wave speed values below 1.2 m/s were neglected yielding holes in the colored SWS overlay. Note, B-mode and elastogram result from different scans and show slightly varying anatomical regions.

**Supplementary table:** description of subjects and measured SWS values. Baseline values were measured in 26 volunteers whereas the effect of the Valsalva maneuver (VM) on brain stiffness was investigated by SWS measurements prior to (SWS_pre_) and during VM (SWS_VM_) in 10 volunteers.

|  |  |  |  |  |  | Baseline values | | Valsalva experiment | |  |  |  |
| --- | --- | --- | --- | --- | --- | --- | --- | --- | --- | --- | --- | --- |
| **# Subject** | **Sex** | **Age** | **Heart rate** | **BP systolic** | **BP diastolic** | **SWS** | **SD** | **SWS_pre_** | **SD** | **SWS_VM_** | **SD** | **ΔSWS** |
|  |  | **in years** | **in bpm** | **in mmHg** | **in mmHg** | **in m/s** | **in m/s** | **in m/s** | **in m/s** | **in m/s** | **in m/s** | **in %** |
| 1 | m | 43 | 66 | 129 | 82 | 1.51 | 0.08 | 1.64 | 0.10 | 1.73 | 0.11 | 5.2 |
| 2 | m | 28 | 63 | 119 | 71 | 1.56 | 0.07 | 1.64 | 0.10 | 1.80 | 0.09 | 8.9 |
| 3 | m | 27 | 55 | 118 | 60 | 1.63 | 0.08 | 1.67 | 0.14 | 1.82 | 0.16 | 8.2 |
| 4 | f | 26 | 53 | 109 | 75 | 1.70 | 0.17 | 1.56 | 0.09 | 1.76 | 0.17 | 11.4 |
| 5 | m | 32 | 59 | 109 | 69 | 1.59 | 0.12 | 1.58 | 0.06 | 1.81 | 0.08 | 12.7 |
| 6 | m | 47 | 60 | 118 | 71 | 1.50 | 0.13 | 1.62 | 0.13 | 1.79 | 0.12 | 9.5 |
| 7 | f | 32 | 81 | 102 | 65 | 1.65 | 0.07 | 1.59 | 0.13 | 1.78 | 0.12 | 10.7 |
| 8 | f | 26 | 62 | 128 | 81 | 1.58 | 0.10 | 1.55 | 0.12 | 1.72 | 0.18 | 9.9 |
| 9 | f | 26 | 60 | 110 | 68 | 1.62 | 0.09 | 1.57 | 0.19 | 1.71 | 0.15 | 8.2 |
| 10 | m | 34 | 70 | 110 | 55 | 1.59 | 0.12 | 1.59 | 0.12 | 1.82 | 0.21 | 12.6 |
| 11 | m | 21 | 62 | 118 | 74 | 1.55 | 0.14 |  |  |  |  |  |
| 12 | m | 22 | 74 | 129 | 75 | 1.67 | 0.14 |  |  |  |  |  |
| 13 | m | 25 | 59 | 120 | 70 | 1.60 | 0.07 |  |  |  |  |  |
| 14 | m | 26 | 55 | 105 | 70 | 1.52 | 0.07 |  |  |  |  |  |
| 15 | m | 29 | 64 | 121 | 62 | 1.62 | 0.10 |  |  |  |  |  |
| 16 | m | 33 | 79 | 130 | 68 | 1.56 | 0.14 |  |  |  |  |  |
| 17 | m | 35 | 80 | 121 | 68 | 1.59 | 0.16 |  |  |  |  |  |
| 18 | m | 48 | 51 | 115 | 67 | 1.52 | 0.05 |  |  |  |  |  |
| 19 | m | 56 | 62 | 174 | 85 | 1.49 | 0.11 |  |  |  |  |  |
| 20 | m | 71 | 56 | 133 | 80 | 1.55 | 0.07 |  |  |  |  |  |
| 21 | m | 74 | 65 | 129 | 70 | 1.53 | 0.07 |  |  |  |  |  |
| 22 | f | 25 | 67 | 109 | 72 | 1.67 | 0.08 |  |  |  |  |  |
| 23 | f | 28 | 51 | 120 | 71 | 1.66 | 0.07 |  |  |  |  |  |
| 24 | f | 64 | 84 | 126 | 78 | 1.40 | 0.07 |  |  |  |  |  |
| 25 | f | 72 | 77 | 125 | 66 | 1.47 | 0.07 |  |  |  |  |  |
| 26 | f | 86 | 92 | 122 | 64 | 1.41 | 0.04 |  |  |  |  |  |
|  | **Mean** | 39.9 | 65.7 | 121.1 | 70.7 | 1.57 | 0.10 | 1.60 | 0.12 | 1.77 | 0.14 | 9.8 |
|  | **Median** | 32 | 63 | 120 | 70 | 1.57 | 0.08 | 1.59 | 0.12 | 1.79 | 0.14 | 9.7 |
|  | **SD** | 18.6 | 10.9 | 13.7 | 6.9 | 0.08 | 0.03 | 0.04 | 0.03 | 0.04 | 0.04 | 2.28 |
|  | **Min** | 21 | 51 | 102 | 55 | 1.40 | 0.04 | 1.55 | 0.06 | 1.71 | 0.08 | 5.2 |
|  | **Max** | 86 | 92 | 174 | 85 | 1.70 | 0.17 | 1.67 | 0.19 | 1.82 | 0.21 | 12.7 |

SD: standard deviation, BP systolic: blood pressure during systole, BP diastolic: blood pressure during diastole
